# Paying for Medical and Social Complexity in Massachusetts Medicaid

**DOI:** 10.1001/jamanetworkopen.2023.32173

**Published:** 2023-09-05

**Authors:** Matthew J. Alcusky, Eric O. Mick, Jeroan J. Allison, Catarina I. Kiefe, Meagan J. Sabatino, Frances E. Eanet, Arlene S. Ash

**Affiliations:** 1 Department of Population and Quantitative Health Sciences, University of Massachusetts Chan Medical School, Worcester

## Abstract

**Importance:**

The first MassHealth Social Determinants of Health payment model boosted payments for groups with unstable housing and those living in socioeconomically stressed neighborhoods. Improvements were designed to address previously mispriced subgroups and promote equitable payments to MassHealth accountable care organizations (ACOs).

**Objective:**

To develop a model that ensures payments largely follow observed costs for members with complex health and/or social risks.

**Design, Setting, and Participants:**

This cross sectional study used administrative data for members of the Massachusetts Medicaid program MassHealth in 2016 or 2017. Participants included members who were eligible for MassHealth’s managed care, aged 0 to 64 years, and enrolled for at least 183 days in 2017. A new total cost of care model was developed and its performance compared with 2 earlier models. All models were fit to 2017 data (most recent available) and validated on 2016 data. Analyses were begun in February 2019 and completed in January 2023.

**Exposures:**

Model 1 used age-sex categories, a diagnosis-based morbidity relative risk score (RRS), disability, serious mental illness, substance use disorder, housing problems, and neighborhood stress. Model 2 added an interaction for unstable housing with RRS. Model 3 added rurality and updated diagnosis-based RRS, medication-based RRS, and interactions between sociodemographic characteristics and morbidity.

**Main Outcome and Measures:**

Total 2017 annual cost was modeled and overall model performance (*R^2^*) and fair pricing of subgroups evaluated using observed-to-expected (O:E) ratios.

**Results:**

Among 1 323 424 members, mean (SD) age was 26.4 (17.9) years, 53.4% were female (46.6% male), and mean (SD) 2017 cost was $5862 ($15 417). The *R^2^* for models 1, 2, and 3 was 52.1%, 51.5%, and 60.3%, respectively. Earlier models overestimated costs for members without behavioral health conditions (O:E ratios 0.94 and 0.93 for models 1 and 2, respectively) and underestimated costs for those with behavioral health conditions (O:E ratio >1.10); model 3 O:E ratios were near 1.00. Model 3 was better calibrated for members with housing problems, those with children, and those with high morbidity scores. It reduced underpayments to ACOs whose members had high medical and social complexity. Absolute and relative model performance were similar in 2016 data.

**Conclusions and Relevance:**

In this cross-sectional study of data from Massachusetts Medicaid, careful modeling of social and medical risk improved model performance and mitigated underpayments to safety-net systems.

## Key Points

**Question:**

Can risk modeling make payments for Medicaid beneficiaries that promote equity by accounting for medical complexity and social determinants of health?

**Findings:**

This cross-sectional study of more than 1.3 million members of Massachusetts Medicaid found that the use of interaction terms better accounted for additional costs associated with caring for members with greater medical and social complexity. The model with interactions improved on earlier versions and allocated payments to health systems more equitably.

**Meaning:**

As equity becomes increasingly important in health care delivery, careful modeling of social and medical risk can improve accuracy, ensuring that payment aligns with elevated needs of vulnerable subgroups.

## Teaser

This cross-sectional study examines administrative data from the Massachusetts Medicaid program MassHealth and develops models to account for caring for members with greater complexity and allocate payments more equitably.

## Introduction

As health care payment shifts from fee-for-service toward managed care, risk adjustment models are used to pay health plans based on the expected costliness of their enrollees.^1,2^ The models are used to equitably distribute a state-determined total budget. Risk-adjusted payments should account for the cost of meeting each individual’s need for health care services, rather than mirror historical, possibly inequitable, spending. The potential for such harm was demonstrated in a research exercise that added race and ethnicity variables to Medicare Advantage risk models.^3^ Modeling decisions must consider equity and transparency, as well as the perverse consequences of mispricing readily identified patient subgroups.

The University of Massachusetts Chan Medical School partners with the Massachusetts Executive Office of Health and Human Services to develop payment models addressing both social determinants of health (SDH) and medical risk factors for MassHealth, Massachusetts’ Medicaid and Children’s Health Insurance Program. MassHealth has used SDH models to inform payments since October 2016^4^; it is still one of few states to do so.^5^

Equitable payment is increasingly important as policymakers and payers pursue system transformation to integrated and accountable models of care. Accountable care organizations (ACOs) represent an increasingly prevalent alternative payment model in which a health system is paid and accepts responsibility for managing an attributed population’s health and bears financial risk associated with cost and quality performance. Health care organizations that lack confidence that payments will cover their costs may choose not to participate in alternative payment models. Organizations that choose to participate despite underpayment may lose money (with downstream consequences for quality and access), try to discourage enrollment from underpriced subgroups of members, or seek to underspend on or undertreat members with high levels of unmet needs, leading to worse health outcomes.^6^

During 2018, MassHealth shifted nearly 1 million members into 17 Medicaid ACOs and replaced their original SDH payment model (model 1), developed on 2013 data, with model 2, developed on 2015 data. Model 3 was built using 2017 data and implemented in January 2020 to address concerns brought to MassHealth by stakeholders (and supported by preliminary analyses) about inadequate payments for high-complexity members with comorbid medical, behavioral, and social risks and the ACOs that disproportionately care for them. With model 3, we sought refinements of the earlier models to mitigate mispricing of clinical and policy-relevant subgroups.

## Methods

Administrative data from MassHealth included member eligibility and enrollment files, encounter records, and billing claims for calendar years 2016 and 2017. Analyses were begun in February 2019 and completed in January 2023. This work, conducted using deidentified data for program operations, was not considered human participants research; thus, our university institutional review board was not involved and consent from MassHealth members not required. We followed the Strengthening the Reporting of Observational Studies in Epidemiology (STROBE) reporting guideline for cross-sectional studies.

### Population

Using a cross-sectional study design, we studied MassHealth’s managed-care–eligible members, aged 0 to 64 years, who were enrolled for at least 183 days in ACOs, managed care organizations, or state-administered managed care in 2017 (or 2016 for model validation). We report the race and ethnicity of those included in the study based on member self-report as recorded in MassHealth administrative data. This information was not used in model development, only to describe the study population and examine how closely the model’s expected values tracked costs for each racial and ethnic group.

### Outcome

We modeled cost, actuarially adjusted (amounts paid by managed care plans were replaced with MassHealth-specified payments), annualized, and then top-coded at $200 000. Actuarial (rate-book) pricing eliminates the presumption that care delivered by more expensive systems is more valuable. All inpatient, outpatient, and pharmacy costs were included, but not long-term services and supports costs, which are excluded from ACO cost accountability benchmarks. Prior work suggests that functional status information, unavailable to us, is required to explain the need for spending on long-term services and supports.^7^

### Explanatory Variables

We did not consider utilization measures as indicators of need, but rather relied on demographic information, a summary medical morbidity score calculated from coded diagnoses using DxCG software^8,9^ and SDH characteristics (eTable 1 in Supplement 1). Utilization measures are correlated with need, but using them in modeling would introduce undesirable incentives for excessive care. In all models, the most powerful explanatory variables are the relative risk scores (RRS) based on diagnoses coded in administrative data (DxCG scores) and calculated using Cotiviti software.^8,9^ Models 1 and 2 used a DxCG RRS (Cotiviti model No. 312) developed on 2012 Medicaid data.^4,8,9^ In model 3, we substituted a diagnosis-based model (No. 88 in the DxCG software suite), calibrated on 2015 commercially insured populations, because it captured the relationship between medical complexity and cost better (adding more than 3 percentage points to explanatory power).^8,9^ We also examined the value of including in model 3 a risk score based on pharmacy data (RxCG model No. 86) developed on the same 2015 commercial data and relying on national drug codes from prescriptions rather than diagnoses. A pharmacy-based model can identify some severity issues not apparent in diagnoses (eg, diabetes management with insulin) and largely avoids bias due to aggressive diagnostic upcoding.

Model 1 has been described previously.^4^ Briefly, it was developed using 2013 fee-for-service data and launched in 2016. It includes the following: 10 age categories (ranging from 0-64 years) separately for each sex, a DxCG (model No. 312) RRS, a disability hierarchy (Department of Mental Health [DMH] client, Department of Developmental Services [DDS] but not a DMH client, entitled to Medicaid due to disability but not a DMH or DDS client, and no disability), behavioral health conditions (serious mental illness [SMI] or substance use disorder [SUD]), housing problems (a Z59.0x code for homelessness or ≥3 addresses in a year), and the Neighborhood Stress Score (NSS), a census-block-group–level measure of socioeconomic stress.^4^ The NSS is standardized to have mean = 0 and SD = 1 across the entire Medicaid population.

Differences among the models are detailed in eTable 2 in Supplement 1. Model 2 was fit using 2015 data and implemented in 2018. It addressed previous underpayments for individuals considered medically complex with housing problems using an interaction term with its DxCG (model No. 312) RRS. We also increased the top-coding threshold from $125 000 in model 1 to $200 000 in model 2 to improve accuracy for high-cost members and adjusted for NSS only when it was positive (ie, NSS+ only adds dollars for neighborhoods with above-average socioeconomic stress). With these changes, model 2 no longer required special indicators for SMI and SUD to properly price members with these conditions.

Medicaid programs insure many children and adolescents. Most children are fairly healthy, and about one-third of all spending on children is used for the most medically complex 1%.^10^ In model 3, we sought to improve accuracy for both healthy and high-acuity youth by adding an interaction between the DxCG score and younger age groups.

Model 3 specifically addresses costs associated with co-occurring behavioral health conditions, medical morbidity, and neighborhood environment. Motivated by MassHealth’s policy focus on behavioral health, we examined narrower subgroups with 1 or more behavioral health conditions. Model 3 includes terms for any SMI without co-occurring opioid use disorder (OUD), OUD without SMI, and OUD with SMI, as each category was associated with increased costs. Terms for other drug use disorders and alcohol use disorders were considered but ultimately not included because the model already captured their costs well. Presence of at least 1 SMI or SUD diagnosis defines the behavioral health category. Model 3 also includes an interaction between the NSS+ and its DxCG (model No. 88) score, recognizing that neighborhood stress is more strongly associated with health care costs as medical complexity rises. Finally, we added a rurality indicator (using Urban Boundaries 2010 from the MassDOT Open Data Portal) to address concerns about rural underservice.^11,12,13^

### Statistical Analysis

Model development was completed in 2019 for MassHealth to implement in 2020. For all models (1, 2, and 3), we used risk factors measured in 2017 (the most recent data available at the time) to model annualized cost in 2017 (concurrent modeling) using least-squares regression weighted by time enrolled. We refit the original structure of the 2 earlier models to 2017 data to fairly compare their performance with that of model 3. All diagnoses in both years of data were coded using *International Statistical Classification of Diseases, Tenth Revision, Clinical Modification (ICD-10-CM)*. We report coefficients for each model with standard errors.

During model development, we considered various interactions between medical and social complexity that could generate larger payments for members with both social risk factors and medical complexity than for the sum of these problems found separately (eMethods in Supplement 1). For example, the final model pays more for members with both housing and behavioral health problems, indexed to the DxCG score (No. 88 in the DxCG software suite)^8,9^; that is, it adds progressively more for those with greater medical morbidity.

We first used 2017 data to evaluate model performance using explanatory power (*R^2^*) and observed-to-expected (O:E) ratios, which divide observed values by model-expected values for subgroups of interest. A good model yields O:E ratios close to 1.0, since deviations from 1 indicate mispricing: ratios less than 1 for a population suggest underpayment and greater than 1, overpayment. We recalculated (validated) these performance measures in 2016 data, the most recent data available on which none of the models had been developed. We developed the model on the most recent data available to reduce the effects of secular changes between development and implementation. When calculating O:E ratios in 2016, we normalized coefficients from the model developed in 2017, to make the E for the whole population match the O of 2016 cost. To explore implications of changes from model 2 to model 3 for MassHealth ACOs, we applied MassHealth’s algorithm to attribute members to ACOs based on their primary care clinician affiliation.

## Results

The 2017 study population included 1 323 424 members (1 280 151 person-years) with a mean (SD) cost of $5862 ($15 417). Mean (SD) age was 26.4 (17.9) years; 53.4% were female and 46.6% male (Table 1). Almost 15% of members had either SMI or OUD, and 13% had housing problems.

**Table 1.** Characteristics of MassHealth Study Population in 2017^a^

Characteristic	Person-years (%)^b^	Mean (SD)		
		DxCG score^c^	RxCG score^d^	Cost^e^
Total	1 280 152 (100.0)	1.0 (2.3)	1.0 (2.3)	5862 (15 417)
Age, mean (SD), y	26.4 (17.9)	NA	NA	NA
Sex				
Male	594 116 (46.4)	0.9 (2.5)	1.0 (2.4)	5709 (16 254)
Female	686 036 (53.6)	1.1 (2.1)	1.1 (2.1)	5995 (14 652)
Race and ethnicity				
Black non-Hispanic	129 535 (10.1)	0.9 (2.3)	0.9 (2.2)	5166 (14 788)
Hispanic	93 734 (7.3)	1.0 (2.3)	1.0 (2.2)	6142 (15 505)
White non-Hispanic	466 737 (36.5)	1.1 (2.4)	1.2 (2.5)	6994 (16 962)
Other non-Hispanic^f^	73 696 (5.8)	0.7 (1.7)	0.6 (1.6)	3669 (11 136)
Missing/unknown	516 451 (40.3)	1.0 (2.3)	0.9 (2.2)	5277 (14 514)
Disability status^g^				
Client of DMH	8610 (0.7)	2.7 (3.2)	2.9 (3.6)	29 689 (37 106)
Client of DDS (not DMH)	18 433 (1.4)	1.6 (3.2)	1.9 (3.6)	14 479 (26 721)
All other disabled	122 312 (9.6)	2.9 (4.7)	3.0 (4.5)	18 937 (31 025)
Not disabled	1 132 779 (88.5)	0.8 (1.7)	0.8 (1.7)	4167 (10 904)
Rural locality	58 433 (4.6)	0.9 (2.0)	0.9 (2.1)	5410 (13 963)
NSS+, mean (SD)^h^	0.4 (0.6)	NA	NA	NA
Housing problems^i^				
No housing problems	1 115 389 (87.1)	0.9 (2.2)	0.9 (2.1)	5248 (14 140)
Any housing problems	164 763 (12.9)	1.4 (3.0)	1.7 (3.2)	10 020 (21 760)
Unstably housed (≥3 addresses)	157 400 (12.3)	1.4 (2.9)	1.5 (3.0)	9048 (20 162)
Homelessness by *ICD* code	13 322 (1.0)	3.4 (4.5)	6.2 (5.5)	33 245 (39 932)
Behavioral health conditions				
No SMI or SUD	1 094 071 (85.5)	0.8 (1.9)	0.7 (1.7)	3804 (11 267)
SMI only	103 558 (8.1)	2.1 (3.0)	2.2 (2.9)	14 363 (22 710)
OUD only	31 280 (2.4)	2.3 (3.5)	2.4 (3.4)	16 585 (24 234)
Other drug use disorder only	5093 (0.4)	2.1 (4.3)	3.1 (4.8)	14 436 (27 856)
AUD only	8845 (0.7)	2.4 (3.9)	3.7 (4.9)	16 611 (28 142)
Dual diagnoses				
SMI and OUD	22 010 (1.7)	3.8 (4.0)	5.1 (4.7)	32 354 (35 076)
SMI and other drug use disorder	8062 (0.6)	2.9 (3.6)	4.8 (4.4)	26 835 (32 929)
SMI and AUD	7233 (0.6)	3.3 (4.0)	5.1 (5.0)	25 971 (32 845)

Abbreviations: AUD, alcohol use disorder; DDS, Department of Developmental Services; DMH, Department of Mental Health; *ICD*, *International Classification of Diseases*; NA, not applicable; NSS, Neighborhood Stress Score; OUD, opioid use disorder; SMI, serious mental illness; SUD, substance use disorder.

^a^ Study population is MassHealth members aged 0 to 64 years, enrolled and eligible for managed care for at least 183 days in 2017. The full population used for normalizing scores includes those enrolled for at least 1 day.

^b^ All mean and percentage values are calculated based on person-time contributed rather than counts of individuals.

^c^ The DxCG score is the DxCG version 5.3 diagnosis-based concurrent risk score (Cotiviti model No. 88), normalized to have mean = 1 in the full population.

^d^ The RxCG score is the pharmacy-based concurrent risk score (Cotiviti model No. 86), normalized to have mean = 1 in the full population.

^e^ Cost is calculated using MassHealth “rate book” prices obtained from MassHealth’s actuaries and is the total cost of MassHealth covered services, excluding spending on long-term services and supports.

^f^ The other race and ethnicity category includes all racial and ethnic groups except for Black non-Hispanic, Hispanic, and White non-Hispanic.

^g^ Disability is coded hierarchically: first, MassHealth eligibility as client of the DMH; otherwise, client of the DDS; if neither DMH nor DDS, entitled to Medicaid because of disability (“All other disabled”).

^h^ Neighborhood Stress Score, normalized to the full population (which includes members enrolled for at least 1 day); NSS+ equals the Neighborhood Stress Score when it is positive and 0 otherwise.

^i^ Housing problems were defined as having 3 or more addresses or being coded as homeless during 2017.

The *R^2^* of model 3 was 60.3%, compared with 51.5% for model 2 and 52.1% for model 1 (Table 2 and eTable 3 in Supplement 1). Gains in explanatory power were largely associated with using the updated No. 88 DxCG RRS and adding the No. 86 RxCG RRS. Adding the RxCG improved explanatory power by 5 percentage points, without increasing the total dollar value associated with medical morbidity measures. Thus, adding the RxCG did not meaningfully change the expected value for an individual of average medical morbidity on both scores but improved accuracy when the 2 scores provided complementary information.

**Table 2.** Coefficients and Explanatory Power of MassHealth Total Cost of Care SDH Risk Adjustment Models 1, 2, and 3 in 2017^a^

Characteristic	Coefficient (SE)		
	SDH model 1	SDH model 2	SDH model 3
*R^2^*, %	52.07	51.49	60.29
20 Age-sex categories^b^	Various	Various	Various
Disability status^c^			
Client of DMH	17 449 (140.8)	15 146 (132.3)	12 604 (105.3)
Client of DDS (not DMH)	3247 (106.7)	782 (86.7)	4251 (71.7)
All other disabled	1806 (50.7)	2460 (37.3)	2200 (31.7)
Rural locality	NA	NA	171 (40.5)
NSS or NSS+^d^	55 (15.9)	17 (10.5)	NA
Housing problems^e^	702 (51.8)	220 (41.2)	NA
Behavioral health conditions			
SMI	2901 (53.5)	NA	NA
SUD	2546 (62.7)	NA	NA
SMI, no OUD	NA	NA	1707 (31.6)
OUD, no SMI	NA	NA	3753 (56.6)
OUD with SMI	NA	NA	5594 (71.0)
RxCG RRS No. 86^f^	NA	NA	1939 (4.8)
DxCG RRS No. 312^g^	4218 (8.2)	5388 (5.6)	NA
DxCG RRS No. 88^g^	NA	NA	3406 (6.0)
DxCG RRS No. 88 × NSS+^d^^,^^g^	NA	NA	26 (5.3)
DxCG RRS No. 88 × age 0-14^g^	NA	NA	390 (12.2)
DxCG RRS No. 88 × age 15-20^g^	NA	NA	636 (20.7)
DxCG RRS No. 312 × housing problems^e^^,^^g^	NA	479 (13.2)	NA
DxCG RRS No. 88 × housing problems × any BHD^e^^,^^g^	NA	NA	592 (10.0)

Abbreviations: BHD, behavioral health diagnosis; DDS, Department of Developmental Services; DMH, Department of Mental Health; NA, not applicable; NSS, Neighborhood Stress Score; OUD, opioid use disorder; RRS, relative risk score; SDH, social determinants of health; SMI, serious mental illness; SUD, substance use disorder.

^a^ Study population is MassHealth members aged 0 to 64 years, enrolled and eligible for managed care for at least 183 days in 2017. The full population used for normalizing scores includes those enrolled for at least 1 day. The models used the following data: model 1 was developed on 2013 data and applied on 2017 data, model 2 was developed on 2015 data and applied on 2017 data, and model 3 was developed and applied on 2017 data.

^b^ Each model also assigns dollars to 20 age-sex categories; see eTable 3 in Supplement 1.

^c^ Disability is coded hierarchically: first, MassHealth eligibility as client of the DMH; otherwise, client of the DDS; if neither DMH nor DDS, entitled to Medicaid due to disability (“All other disabled”).

^d^ Neighborhood Stress Score, normalized to the full population; NSS+ equals the Neighborhood Stress Score when it is positive and 0 otherwise.

^e^ Housing problems were defined as having 3 or more addresses or being coded as homeless during 2017.

^f^ The RxCG score is the pharmacy-based concurrent risk score (Cotiviti version 5.3, model No. 86), normalized to have mean = 1 in the full population.

^g^ In models 1 and 2, the DxCG RRS is the DxCG version 4.2 diagnosis-based concurrent risk score (Cotiviti model No. 312). In model 3, the DxCG RRS is the DxCG version 5.3 diagnosis-based concurrent risk score (Cotiviti model No. 88). Both RRS are normalized to have mean = 1 in the full population.

Other modifications to model 3 improved calibration for several sociodemographic and clinical subgroups of MassHealth members (Table 3). Models 1 and 2 overestimated costs for members without behavioral health conditions (O:E ratio 0.94 and 0.93, respectively) and underestimated them for those with OUD (O:E ratio 1.41 and 1.42, respectively) and dual SMI-OUD diagnoses (O:E ratio 1.29 and 1.29, respectively). Model 3–expected values for members with OUD, SMI, or both were within 1% of observed costs (ie, O:E ratio range 0.99-1.01); expected values for members with unstable housing and homelessness were also more accurate.

**Table 3.** Observed-to-Expected Ratios for MassHealth Total Cost of Care SDH Risk Adjustment Models in 2017^a^

Characteristic	Cost, $	O:E ratio		
		Model 1 (*R^2^* = 52.07%)	Model 2 (*R^2^* = 51.49%)	Model 3 (*R^2^* = 60.29%)
Total	5862	NA	NA	NA
Age, y				
0-14	3104	0.85	1.04	1.02
15-20	3391	0.87	1.00	0.97
21-64	8049	1.06	0.99	1.00
Sex				
Male	5995	0.97	0.99	1.00
Female	5709	1.05	1.01	0.99
Race and ethnicity				
Black non-Hispanic	5166	0.94	0.94	0.96
Hispanic	6142	1.01	1.02	1.01
White non-Hispanic	6994	1.06	1.04	1.03
Other non-Hispanic^b^	3669	0.94	0.98	1.03
Missing/unknown	5277	0.96	0.96	0.98
Disability status^c^				
Client of DMH	29 689	0.87	0.91	1.01
Client of DDS (not DMH)	14 479	1.03	1.11	1.01
All other disabled	18 937	1.15	1.01	1.01
Not disabled	4167	0.94	0.99	1.00
Rural locality	5410	1.01	1.01	1.00
NSS top 10%^d^	6486	1.01	1.01	1.00
NSS bottom 10%^d^	5703	1.02	1.01	1.03
Housing problems^e^				
No housing problems	5248	1.00	1.01	1.01
Any housing problems	10 020	1.00	0.96	0.98
Unstably housed (≥3 addresses)	9048	0.97	0.93	0.97
Homelessness by *ICD* code	33 245	1.20	1.05	0.99
Behavioral health conditions				
No SMI or SUD	3804	0.94	0.93	1.00
SMI only	14 363	0.96	1.02	1.00
OUD only	16 585	1.41	1.42	1.00
Other drug use disorder only	14 436	0.94	0.87	0.95
AUD only	16 611	1.02	0.89	0.97
Dual diagnoses				
SMI and OUD	32 354	1.29	1.29	1.00
SMI and other drug use disorder	26 835	1.01	1.01	1.02
SMI and AUD	25 971	1.01	0.99	0.98
DxCG score^f^				
1-90th Percentile	3038	0.87	1.01	1.01
90-94th Percentile	15 900	1.15	1.09	1.01
95-97th Percentile	23 867	1.20	1.05	0.97
98-100th Percentile	54 326	1.13	0.91	0.99

Abbreviations: AUD, alcohol use disorder; DDS, Department of Developmental Services; DMH, Department of Mental Health; *ICD*, *International Classification of Diseases*; NA, not applicable; NSS, Neighborhood Stress Score; O:E, observed to expected; OUD, opioid use disorder; SDH, social determinants of health; SMI, serious mental illness; SUD, substance use disorder.

^a^ The mean cost in 2017 was $5862 (top-coded at $200 000 and annualized). Model-expected values were made from SDH models 1 through 3. Model-expected values were first bottom- and top-coded ($60-$200 000) and then standardized to study-population 2017 mean costs to calculate O:E ratios (which divide observed values by model-expected values for subgroups of interest). A good model yields O:E ratios close to 1.0, since O:E ratios less than 1 indicate populations for which expected costs exceed observed costs and O:E ratios greater than 1, underpricing compared to observed costs. O:E ratios are not subject to sampling error.

^b^ The other race and ethnicity category includes all racial and ethnic groups except for Black non-Hispanic, Hispanic, and White non-Hispanic.

^c^ Disability is coded hierarchically: first, MassHealth eligibility as client of the DMH; otherwise, client of the DDS; if neither DMH nor DDS, entitled to Medicaid because of disability (“All other disabled”).

^d^ Neighborhood Stress Score, normalized to the full population (which includes members enrolled for at least 1 day); NSS+ equals the Neighborhood Stress Score when it is positive and 0 otherwise.

^e^ Housing problems were defined as having 3 or more addresses or being coded as homeless during 2017.

^f^ The DxCG score for models 1 and 2 is the DxCG version 4.2 Cotiviti model No. 312 diagnosis-based concurrent risk score normalized to have mean = 1 in the full population, and the DxCG score for model 3 is the DxCG version 5.3 Cotiviti model No. 88, normalized to have mean = 1 in the full population.

Model 3 was better calibrated for members with the highest medical morbidity. Model 1 substantially overestimated costs for the 90% of members with the least medical complexity (lowest model No. 312 DxCG scores) and underestimated for those with the highest such scores. Model 2 was better calibrated but still underestimated for those in the 90th to 94th percentile of the (No. 312 model) DxCG RRS (O:E ratio 1.09) and overestimated for those in the 98th to 100th percentile (O:E ratio 0.91). Model 3–expected values were within 3% of observed values for each of these groups. Model 3 also recognized costs of $3404 for each unit of its (No. 88 model) DxCG RRS for adults but $3800 for children (aged 0-14 years) and $4030 for adolescents (aged 15-20 years).

In model 3, residence in a rural area was associated with $171 additional cost, addressing a modest underpayment in model 2 (O:E ratio 1.01). Members whose NSS+ was 1 SD above the mean were expected to cost an additional $23 per unit of the DxCG RRS (eg, $230 for a member with NSS+ = 1 and RRS = 10). Model 3 also contained an extra payment for people with housing and behavioral health problems in an interaction with its DxCG RRS. The coefficient of $503 associated with this interaction would, for example, add a little more than $2000 for a member who was homeless with OUD and a DxCG score of 4.

When applied to 2016 data, the explanatory power for model 3 was similar (60.1% vs 60.3%) to its performance in the development data and greater than for models 1 and 2. Expected costs were within 10% of observed costs for all subgroups examined and usually much closer (eTable 4 in Supplement 1).

### ACO Results

To illustrate the implications of changes implemented in SDH model 3 for ACOs with diverse memberships, we present member characteristics and O:E ratios for illustrative ACOs (Table 4). For example, costs for ACO D, which had above average medical risk (No. 88 DxCG RRS: 1.18) and social risk (18% housing problems, NSS+: 0.58, 11% SUD), were 21% above average. However, the observed costs of ACO D exceeded model 2 expectations by only 6% and model 3 expectations by 3%. The difference between model 2 and 3 expectations was $268 per person. For a typical ACO with about 40 000 members (median, 36 480), this difference translates to more than $10 million.

**Table 4.** Observed-to-Expected Total Cost of Care in 2017 for Selected Attributed ACOs by Model^a^

Member characteristics						No adjustment		Risk adjustment model			
ACO	Size^b^	No. 88 DxCG RRS, mean^c^	Housing problem, %^d^	NSS+, mean^e^	SUD, %	Observed		SDH model 2		SDH model 3	
						Cost, $	O:E ratio	Expected cost, $	O:E ratio	Expected cost, $	O:E ratio
All	1.3 million	1.01	13.0	0.43	8.0	5862	1.00	5862	1.00	5862	1.00
A	Small	1.05	14.4	0.56	9.0	5299	0.90	6030	0.88	6031	0.87
B	Small	0.93	9.4	0.20	6.0	4999	0.85	5362	0.93	5116	0.96
C	Small	1.18	12.8	0.26	14.0	7011	1.20	6620	1.06	6793	1.04
D	Large	1.18	18.0	0.58	11.0	7103	1.21	6704	1.06	6972	1.03
E	Large	1.03	8.8	0.31	6.0	5673	0.97	5802	0.98	5638	1.02
F	Large	1.09	19.8	1.10	8.0	6682	1.14	6590	1.01	6730	0.99

Abbreviations: ACO, accountable care organization; NSS, Neighborhood Stress Score; O:E, observed to expected; RRS, relative risk score; SDH, social determinants of health; SUD, substance use disorder.

^a^ The mean cost in 2017 was $5862 (top-coded at $200 000 and annualized). Model-expected values were first bottom- and top-coded ($60-$200 000) and then standardized to 2017 mean costs to calculate O:E ratios in the study population; O:E ratios are not subject to sampling error.

^b^ ACOs are small if they had 2017 enrollment below the median (36 480 person-years); else, they are large.

^c^ DxCG RRS is the DxCG version 5.3 diagnosis-based concurrent risk score (Cotiviti model No. 88), normalized to have mean = 1 in the ever-enrolled managed-care–eligible (full) MassHealth population in 2017.

^d^ Housing problems were defined as having 3 or more addresses or being coded as homeless during 2017.

^e^ Neighborhood Stress Score, normalized to the full MassHealth population; NSS+ equals the Neighborhood Stress Score when it is positive and 0 otherwise.

## Discussion

Equitable payment is fundamental for integrated care models seeking to transform health care by paying for value, not volume. By using interactions to better account for additional costs associated with caring for members with greater medical and social complexity, our new model improves on earlier versions and allocates ACO payments more equitably.

Several SDH risks matter more for members as medical risk increases. NSS+ recognizes increased health care costs associated with contextual social stressors, especially in interaction with the DxCG score. Including an interaction between housing problems with any behavioral health condition and the DxCG score improved accuracy for both homeless individuals and those who are unstably housed. Only model 3 fully recognizes the costs of people experiencing homelessness over those who are merely unstably housed, by accounting for their excess prevalence of behavioral health problems and greater medical morbidity. Consistent with prior literature, we found that housing problems increase health care costs most for people with behavioral health issues and chronic medical conditions.^14,15^

Rurality was associated with higher than expected costs. Although rural residents compose only about 5% of MassHealth members, the $171 rurality coefficient adds about $1.7 million to revenue for an ACO serving 10 000 rural residents. While rural communities are often socioeconomically stressed, so are many urban neighborhoods. The payment for rurality recognizes the extra burden not already captured by the NSS+. Costs of health care inputs (eg, labor) also vary geographically; thus, MassHealth uses actuarially developed geographic rate cell adjustments to align model-predicted payments with regional costs.

Our finding that costs increase with greater social risk may be due to Massachusetts being unusually open to fully treating health problems identified in low-income and other vulnerable populations. Models fit to data in other states or in other settings may find social risk factors associated with lower spending. When this happens, a negative coefficient can be replaced by a positive one so as not to allow payments to replicate what is almost certainly evidence of underspending on vulnerable populations, rather than lesser need. Importantly, our cost outcome was actuarially priced to a common standard, eliminating the bonus that would otherwise flow to more expensive health care delivery systems, which differentially serve wealthier populations. Indeed, the Massachusetts attorney general’s 2022 cost trends report^16^ flags differences in prices (ie, insurance premiums) as a mechanism whereby risk adjustment “may entrench barriers to care and divert funds away from low-income communities.”^17^ That report illustrates how underservice can be quantified and potentially addressed.

Modeling that incorporates social risk factors can help break the link between historical inequities and payments, but intentional reallocations, rather than simply accepting the coefficients that emerge from modeling, may be needed. We check for inequities whenever data allow. For example, expected values for racial and ethnic subgroups (in the approximately 60% of members for whom these data are known) were within ±4% of observed costs. The O:E ratio of 0.96 for Black non-Hispanic members reflects modest overpayment, that is, payments greater than historical cost.

There is always a tension between model accuracy and avoiding establishing perverse incentives. For example, while diagnosis-based models typically describe morbidity burden well, they can drive organizations to invest in upcoding more than improving care.^18^ Model 3 uses the RxCG both to improve accuracy by identifying medical issues and levels of severity that may be inadequately captured with diagnostic codes and to modestly reduce the reward for upcoding diagnoses, since payments for medical morbidity are jointly accounted for by the DxCG and RxCG scores. However, pharmacy-based measures are also imperfect and could be manipulated through changes in prescribing. The federal risk adjustment program has used prescription-based information since 2018 but recognizes fewer diagnosis and pharmacy categories than Cotiviti’s software.^8,9,19,20^

### Limitations

Upcoding and undercoding can unfairly affect plan payments, but this is a limitation of any model that uses administrative data to measure morbidity. MassHealth implemented large-scale delivery system reforms beginning in 2018, suggesting the need for validating and updating the current model with post-reform and post–COVID-19 data. This work is underway. Additional work is needed to quantify the effect of risk-adjustment approaches on ACO success against policy benchmarks, such as achieving shared savings. Discontinuities in the coded prevalence of many disease categories at the 2015 transition from *ICD-9* to* ICD-10* have also been documented,^21^ and the DxCG and RxCG scores used in model 3 were calibrated on data that included only 3 months of *ICD-10* coding.

## Conclusions

Payments that recognize the excess needs of patients with medical and social risks enable safety-net clinicians to participate in payment systems that promote integrated, high-quality care. With thoughtful modeling that includes interactions of medical risk with social risk and other demographics, payments can be better calibrated to need for many disadvantaged groups. MassHealth’s version 3 model recognizes higher levels of need for individuals with comorbid medical and social risks better than earlier models, further mitigating tens of millions of dollars in underpayments each year to Medicaid ACOs serving members with the most complex health care needs.

## Notes

Supplement 1. **eMethods.** Modeling Approach **eTable 1.** Variable Operational Definitions **eTable 2.** Summary of Changes between MassHealth SDH Models 1, 2, and 3 **eTable 3.** Coefficients Including Age-Sex and Intercept Terms and Explanatory Power of MassHealth SDH Total Cost of Care Risk Adjustment Models 1, 2, and 3 in CY2017 **eTable 4.** Observed-to-Expected Ratios for MassHealth SDH Total Cost of Care Risk Adjustment Models 1, 2, and 3 in 2016

Supplement 2.
Data Sharing Statement
