# Supplementary material for: Paying for Medical and Social Complexity in Massachusetts Medicaid
Source: JAMA Netw Open. 2023 Sep 5;6(9):e2332173. doi: 10.1001/jamanetworkopen.2023.32173 (PMC10481227; doi:10.1001/jamanetworkopen.2023.32173)
Supplement: Supplement 1. — eMethods. Modeling Approach eTable 1. Variable Operational Definitions eTable 2. Summary of Changes between MassHealth SDH Models 1, 2, and 3 eTable 3. Coefficients Including Age-Sex and Intercept Terms and Explanatory Power of MassHealth SDH Total Cost of Care Risk Adjustment Models 1, 2, and 3 in CY2017 eTable 4. Observed-to-Expected Ratios for MassHealth SDH Total Cost of Care Risk Adjustment Models 1, 2, and 3 in 2016 [file jamanetwopen-e2332173-s001.pdf]

Supplemental Online Content

Alcusky MJ, Mick EO, Allison JJ, et al. Paying for medical and social complexity in Massachusetts Medicaid. *JAMA Netw Open*. 2023;6(9):e2332173. doi:10.1001/jamanetworkopen.2023.32173

eMethods. Modeling Approach

eTable 1. Variable Operational Definitions

eTable 2. Summary of Changes between MassHealth SDH Models 1, 2, and 3

eTable 3. Coefficients Including Age-Sex and Intercept Terms and Explanatory Power of MassHealth SDH Total Cost of Care Risk Adjustment Models 1, 2, and 3 in CY2017

eTable 4. Observed-to-Expected Ratios for MassHealth SDH Total Cost of Care Risk Adjustment Models 1, 2, and 3 in 2016

This supplemental material has been provided by the authors to give readers additional information about their work.

© 2023 Alcusky MJ et al. *JAMA Network Open.*

eMethods: Modeling Approach

Our modeling approach for social determinants of health (SDH) Model 3 was to begin with Model 2, which was currently in use by MassHealth, and to then examine incremental changes motivated by good modeling practices, newly available data and variables, and policy considerations. Simply refitting Model 2, developed on 2015 data, to the more recent 2017 data produced a small increase (0.3%) in explanatory power. This refitted version of Model 2 (R^2=51.5%) provided a baseline against which the following subsequent changes to the model could be compared.

As summarized in the text of the manuscript and in Supplemental Table 1, most of the gains in explanatory power associated with the transition from Model 2 to Model 3 were obtained with the first few modeling revisions including substituting an updated DxCG medical morbidity summary score, adding a pharmacy based medical morbidity score (the RxCG), and adding terms for serious mental illness (SMI) and substance use disorder (SUD).

Subsequent modeling revisions were made primarily to address concerns regarding the underpricing of specific subgroups of members that were identified by MassHealth as potentially vulnerable, costly, and distributed unevenly across MassHealth ACOs, therein presenting substantial concerns regarding equity if mispriced. These subgroups included youth with medical complexity, members with behavioral health conditions including comorbid SMI and SUD, and members with comorbid medical, behavioral and social risk factors (e.g., homelessness, unstable housing, high neighborhood stress scores).

In Model 3, in line with MassHealth's commitment to payments that support delivery system reforms integrating physical and behavioral health care, we examined more deeply the various relationships among behavioral health conditions and costs. We worried that making distinctions among finer categories of SMI (beyond those differences captured by the summary risk scores) would be overly susceptible to coding manipulation ("gaming"). Thus, we focused on SUD, finding that opioid use disorders (OUD), but not alcohol use disorders, were associated with increased costs. We also sought to improve accuracy for high acuity members, especially youth. Although all our models are constrained to accurately model mean cost within each age-sex category, we suspected that overpayments for healthy children were being balanced against underpayments for sick ones. This concern was acute because of MassHealth's recent launch of 17 Medicaid ACOs with risk-based contracts, and medically complex youth were unevenly distributed across ACOs. Therefore, we explored interaction terms between pediatric age groups and the DxCG risk score.

© 2023 Alcusky MJ et al. *JAMA Network Open.*

We were concerned that individuals with both medical and social risk factors used more resources than those factors individually account for, focusing on interactions of social risk factors with medical morbidity, specifically, the DxCG score. For example, we expect higher costs for a person with mental health issues and no stable place to live, but even more excess costs in the presence of multiple medical problems. Similarly, neighborhood disadvantage can make chronic conditions more difficult to manage, for example, when pollution and low-quality housing exacerbate asthma in children, making hospitalizations more likely. Thus, in Model 3, payments for homelessness or unstable housing, when there is also a behavioral health problem, are indexed to (that is, interacted with) the DxCG score, as are payments associated with neighborhood stress. These interactions generate larger payments for members with both social risk factors and high medical acuity than for these problems individually.

Incremental model adjustments were mostly guided by examination of observed-to-expected ratios (i.e., the ratio of observed costs to model expected costs) for subgroups defined by demographic (age, sex, race/ethnicity, rurality), medical (e.g., percentiles of the DxCG score), behavioral (i.e., SMI, SUD, and component diagnoses), and social characteristics. Other changes were made in pursuit of simplicity and parsimony, and in the case of the age-sex category indicators, the initial decision to remove them was reversed in light of subsequent evidence of mispricing of certain age-sex categories without the indicators.

Policy considerations also informed modeling decisions. Members with dual SMI-SUD comprised only 3% of the population, while those with any behavioral health condition made up nearly 15% of the population. Recognizing the challenge of caring for individuals with social and medical risk factors is made more difficult by the presence of any behavioral health condition, and since differences in performance were not meaningfully different between models, we chose the model with a three-way interaction of housing problems and DxCG score with any behavioral health condition rather than only those with dual SMI-SUD. Finally, due to the real-world problems with retaining negative coefficients in payment models, whereby health systems would be incentivized to under-code an individual's characteristics, model grooming was performed to remove negative coefficients.

© 2023 Alcusky MJ et al. *JAMA Network Open.*

eTable 1: Variable Operational Definitions

Variable	Description and Codes
DxCG Medicaid #312 RRS	DxCG v4.2, Model 312, Medicaid $200k top-coded diagnosis-based model of concurrent total cost of care.
DxCG Commercial #88 RRS	DxCG v5.3, Model 88, Commercial $250k top-coded diagnosis-based model of concurrent total cost of care
RxCG Commercial #86 RRS	RxCG v5.3, Model 86, Commercial $250k top-coded prescription-based model of concurrent total cost of care.
SMI	Serious mental illness defined by Hierarchical Condition Categories (HHS-HCC) 160-163, 166, 168
SUD	Substance use disorders defined by Hierarchical Condition Categories (HHS-HCC) 148-154
Behavioral Health	Diagnosis of SMI or SUD as defined by HHS-HCCs above.
Housing Problems	Unstably housed OR homeless.
Unstably housed (3+ addresses)	Unstably housed defined as more than three addresses in one year.
Homelessness by ICD Code	Homelessness according to ICD10 diagnosis codes.
Disability Status	Disability status is a hierarchy. At the top is enrollment as client of the Department of Mental Health (DMH); else, as a client of the Department of Developmental Services (DDS); else, as entitled to Medicaid due to disability (all other disabled).
NSS+	Neighborhood Stress Score (NSS), normalized to the MassHealth population; NSS+ equals the Neighborhood Stress Score when it is positive and 0 otherwise.
Rural Area	Rural areas identified using the Urban Boundaries 2010 from the MassDOT Open Data Portal
Opioid Use Disorder	ICD10 diagnosis codes:<br>F1110 F11120 F11121 F11122 F11129 F1114 F11150 F11151 F11159 F11181 F11182 F11188 F1119 F1120 F11220 F11221 F11222 F11229 F1123 F1124 F11250 F11251 F11259 F11281 F11282 F11288 F1129<br>ICD9 diagnosis codes:<br>304 30401 30402 30403 3047 30471 30472 30473 3055 30551 30552 30553
Alcohol Use Disorder	ICD10 : F1010 F10120 F10121 F10129 F1014 F10150 F10151 F10159 F10180 F10181 F10182 F10188 F1019 F1020 F10220 F10221 F10229 F10230 F10231 F10232 F10239 F1024 F10250 F10251 F10259 F1026 F1027 F10280 F10281 F10282 F10288 F10<br>ICD 9: 291 2911 2912 2913 2914 2915 29181 29182 29189 2919 303 30301 30302 30303 3039 30391 30392 30393 305 30501 30502 30503;

© 2023 Alcusky MJ et al. *JAMA Network Open.*

Variable	Description and Codes
Other Substance Use Disorder	ICD 10: F1210 F12120 F12121 F12122 F12129 F12150 F12151 F12159 F12180 F12188 F1219 F1220 F12220 F12221 F12222 F12229 F12250 F12251 F12259 F12280 F12288 F1229 F1310 F13120 F13121 F13129 F1314 F13150 F13151 F13159 F13180 F13181 F13182 F13188 F1319 F1320 F13220 F13221 F13229 F13230 F13231 F13232 F13239 F1324 F13250 F13251 F13259 F1326 F1327 F13280 F13281 F13282 F13288 F1329 F1410 F14120 F14121 F14122 F14129 F1414 F14150 F14151 F14159 F14180 F14181 F14182 F14188 F1419 F1420 F14220 F14221 F14222 F14229 F1423 F1424 F14250 F14251 F14259 F14280 F14281 F14282 F14288 F1429 F1510 F15120 F15121 F15122 F15129 F1514 F15150 F15151 F15159 F15180 F15181 F15182 F15188 F1519 F1520 F15220 F15221 F15222 F15229 F1523 F1524 F15250 F15251 F15259 F15280 F15281 F15282 F15288 F1529 F1610 F16120 F16121 F16122 F16129 F1614 F16150 F16151 F16159 F16180 F16183 F16188 F1619 F1620 F16220 F16221 F16229 F1624 F16250 F16251 F16259 F16280 F16283 F16288 F1629 F1810 F18120 F18121 F18129 F1814 F18150 F18151 F18159 F1817 F18180 F18188 F1819 F1820 F18220 F18221 F18229 F1824 F18250 F18251 F18259 F1827 F18280 F18288 F1829 F1910 F19120 F19121 F19122 F19129 F1914 F19150 F19151 F19159 F1916 F1917 F19180 F19181 F19182 F19188 F1919 F1920 F19220 F19221 F19222 F19229 F19230 F19231 F19232 F19239 F1924 F19250 F19251 F19259 F1926 F1927 F19280 F19281 F19282 F19288 F1929;<br>ICD 9: 292 29211 29212 2922 29281 29282 29283 29284 29285 29289 2929 3041 30411 30412 30413 3042 30421 30422 30423 3043 30431 30432 30433 3044 30441 30442 30443 3045 30451 30452 30453 3046 30461 30462 30463 3048 30481 30482 30483 3049 30491 30492 30493 3052 30521 30522 30523 3053 30531 30532 30533 3054 30541 30542 30543 3056 30561 30562 30563 3057 30571 30572 30573 3058 30581 30582 30583 3059 30591 30592 30593

© 2023 Alcusky MJ et al. *JAMA Network Open.*

eTable 2. Summary of Changes between MassHealth SDH Models 1, 2, and 3

	Model 1 (2016)	Model 2 (2018)	Model 3 (2020)
**Data used for model building**	**CY13** state administered primary care managed fee-for-service (FFS) enrollees	**CY15** managed-care-eligible enrollees	**CY17** managed-care-eligible enrollees
**Model launch year**	2016	2018	2020
**Outcome**	Total cost of care, excluding LTSS, top-coded at $125k	Total cost of care, excluding LTSS, top-coded at $200k	Total cost of care, excluding LTSS, top-coded at $200k
**Medical morbidity summary scores**	DxCG (312)<br>Medicaid concurrent model	DxCG (312)<br>Medicaid concurrent model	DxCG (86) and RxCG (88) commercial concurrent models
**SDH variables**	Disability status<br>Behavioral health<br>NSS<br>Housing problems	Disability status<br>NSS+<br>Housing problems	Disability status<br>Behavioral health<br>Opioid use disorder<br>NSS+<br>Rurality<br>Housing problems
**Interactions**	None	Housing problems interacted with DxCG	Multiple SDH variables interacted with DxCG

Abbreviations: long-term services and supports (LTSS); neighborhood stress score (NSS); social determinants of health (SDH).

© 2023 Alcusky MJ et al. *JAMA Network Open.*

eTable 3. Coefficients Including Age-Sex and Intercept Terms and Explanatory Power of MassHealth SDH Total Cost of Care Risk Adjustment Models 1, 2, and 3 in CY2017

	SDH Model 1		SDH Model 2		SDH Model 3	
R-squared	$R^2 = 52.1\%$		$R^2 = 51.5\%$		$R^2 = 60.3\%$	
	Coefficient (95% CI)	SE	Coefficient (95% CI)	SE	Coefficient (95% CI)	SE
Intercept	1,396	71.0	375	49.3	-112	39.6
Disability status [h]						
Client of DMH [e]	17,449	111.4	15,146	114.0	12,510	105.3
Client of DDS f (not DMH)	3,247	84.4	782	74.7	4,224	71.7
All other disabled	1,806	40.1	2,460	32.2	2,185	31.7
Rural locality	NA	NA	NA	NA	171	40.5
NSS7 or NSS+ [e]	55	12.6	NA	NA	NA	NA
Housing problems	702	41.0	220	35.5	NA	NA
BH conditions						
SMI	2,901	42.4	NA	NA	NA	NA
SUD	2,546	49.6	NA	NA	NA	NA
SMI [a] - no OUD [b]	NA	NA	NA	NA	1,707	31.6
OUD - no SMI	NA	NA	NA	NA	3,753	56.6
OUD with SMI	NA	NA	NA	NA	5,594	71.0
RxCG [c]	NA	NA	NA	NA	1,939	4.8
DxCG [d]	4,218	6.5	5,331	5.2	3,406	6.0
DxCG X NSS+ [g]	NA	NA	18	9.1	26	5.3
DxCG X Age 0-14	NA	NA	NA	NA	390	12.2
DxCG X Age 15-20	NA	NA	NA	NA	636	20.7
DxCG X Housing problems	NA	NA	479	11.4	NA	NA
DxCG X Housing problems [h] X any BH [i]	NA	NA	NA	NA	592	10.0
**Age-sex groupings**						
0-1 Female	131	138.2	367	92.0	-1,275	78.2
2-5 Female	Reference		Reference		Reference	
6-12 Female	-349	91.0	-179	61.5	498	49.4
13-17 Female	-225	97.9	-216	67.6	199	55.0
18-24 Female	-497	100.6	-538	65.0	-259	52.3
25-34 Female	-438	93.2	-418	59.7	-293	48.1
35-44 Female	-1,044	96.6	-803	63.4	-756	51.5
45-54 Female	-920	99.5	-498	64.9	-872	53.8
55-59 Female	-772	126.6	-397	79.4	-836	65.9
≥60 Female	-788	129.9	-471	82.7	-984	68.0
0-1 Male	231	135.6	289	91.1	-1,559	77.8
2-5 Male	544	99.0	330	68.5	404	55.1
6-12 Male	199	89.4	-32	60.9	799	49.1
13-17 Male	-488	95.8	-575	66.8	225	54.3
18-24 Male	-2,127	102.7	-1,085	67.5	-615	53.9
25-34 Male	-1,451	98.0	-210	64.5	-497	51.8
35-44 Male	-1,389	100.2	-443	68.5	-480	55.6
45-54 Male	-1,614	97.8	-338	67.2	-869	55.8
55-59 Male	-1,966	125.1	131	83.3	-1,203	68.7
≥60 Male	-1,842	137.6	159	88.9	-1,442	72.9

© 2023 Alcusky MJ et al. *JAMA Network Open.*

Notes: Analyses are weighted (WGT = fraction of the year during which the member was eligible) and include only those enrolled for at least 183 days in 2017. Cost is calculated using "rate book" prices from the actuaries and excludes long-term services and supports (LTSS) spending. Cost is top-coded at $200,000 and annualized = minimum of dollars spent/WGT or $200k. NA = not applicable.

[a] SMI = serious mental illness

[b] OUD = opioid use disorder

[c] RxCG = pharmacy-based concurrent risk score (Cotiviti, Inc., Model 86) normalized to have mean = 1 in the MassHealth population.

[d] The DxCG score for Models 1 and 2 is the DxCG v4.2 Cotiviti, Inc., Model 312 diagnosis-based concurrent risk score normalized to have mean = 1 in the full population and the DxCG score for Model 3 is the DxCG v5.3 (Cotiviti, Inc., Model 88), normalized to have mean = 1 in the full population.

[e] DMH = Mass. Dept. of Mental Health

[f] DDS = Mass. Dept. of Developmental Services

[g] NSS = Neighborhood Stress Score, normalized to the MassHealth population; NSS+ equals the Neighborhood Stress Score when it is positive and 0 otherwise.

[h] Housing problems = 3 or more addresses or coded as homeless during 2017

[i] BH = Behavioral Health

© 2023 Alcusky MJ et al. *JAMA Network Open.*

eTable 4. Observed-to-Expected Ratios for MassHealth SDH Total Cost of Care Risk Adjustment Models 1, 2, and 3 in 2016 [a]

	Person-Years	Cost ($)	Ratio of Group Cost to Population Mean	Model 1 R^2=50.17%	Model 2 R^2=50.96%	Model 3 R^2=59.37%
**Total**	976,752	4,942	**-**	**-**	**-**	**-**
**Characteristic**				**O:E**	**O:E**	**O:E**
Female	461,586	4,760	0.96	1.04	1.01	0.99
Male	515,166	5,105	1.03	0.97	1.00	1.01
Age (years)						
0-14	325,888	2,335	0.47	0.82	1.00	0.95
15-20	118,791	2,895	0.59	0.86	0.98	0.96
21-64	532,073	6,996	1.42	1.07	1.00	1.02
Race/Ethnicity						
White/Non-Hispanic	456,084	6,198	1.25	1.04	1.03	1.02
Black/Non-Hispanic	50,117	4,891	0.99	0.97	0.95	0.98
Hispanic	33,426	4,588	0.93	0.99	1.01	1.01
Other Non-Hispanic	31,082	2,837	0.57	0.97	1.00	1.04
Missing/unknown	406,044	3,728	0.75	0.94	0.96	0.97
Disability status [b]						
Client of DMH	6,559	26,416	5.35	0.94	0.96	1.08
Client of DDS (not DMH)	15,157	11,208	2.27	0.98	1.05	0.97
All other disabled	102,797	15,265	3.09	1.17	1.02	1.02
Not disabled	852,240	3,420	0.69	0.93	0.99	0.99
Rural locality						
Rural	37,612	4,671	0.95	1.01	1.02	1.01
NSS top 10% [c]	98,899	5,374	1.09	0.99	1.00	0.98
NSS bottom 10%	96,911	4,800	0.97	1.03	1.02	1.04
Housing problems						
No housing problems	835,937	4,626	0.94	1.00	1.01	1.00
Any housing problems	140,815	6,819	1.38	0.99	0.97	1.00
Unstably housed (3+ addresses)	138,517	6,406	1.30	0.97	0.96	0.99
Homelessness by ICD code	5,455	28,887	5.85	1.29	1.14	1.10
BH conditions [d]						
No SMI or SUD	796,097	2,947	0.60	0.96	0.95	1.00
SMI only	115,529	11,396	2.31	0.96	1.00	1.00
Opioid use disorder only	20,395	11,930	2.41	1.38	1.41	0.99
Other drug use disorder only	3,075	11,661	2.36	0.96	0.89	0.99
Alcohol use disorder only	5,211	11,708	2.37	0.96	0.87	0.96
Dual Dx SMI + OUD	21,551	23,478	4.75	1.20	1.19	0.97
Dual Dx SMI + other drug use disorder	7,240	22,260	4.50	1.04	1.03	1.05
Dual Dx SMI + AUD	7,656	20,541	4.16	0.98	0.95	0.98
DxCG score [e]			0.00			
1-90th percentile	869,837	2,557	0.52	0.84	0.95	0.96
90-94th percentile	48,469	13,766	2.79	1.11	1.04	1.04
95-97th percentile	29,446	20,761	4.20	1.22	1.09	1.03

© 2023 Alcusky MJ et al. *JAMA Network Open.*

98-100th percentile	29,001	45,663	9.24	1.25	1.02	1.05

[a] The mean cost in CY16 was $4942 (top-coded at 200,000 and annualized). Expected values were made from SDH Models 1-3. Those expectations were top and bottom coded ($60-200,000) and then standardized to the CY16 mean costs to calculate the observed to expected (O:E) ratios.

[b] Disability status indicates MassHealth eligibility as client of the Department of Mental Health (DMH), Department of Developmental Services (DDS), or entitled to Medicaid due to disability ("All other disabled").

[c] NSS = Neighborhood Stress Score, normalized to the MassHealth population; NSS+ equals the Neighborhood Stress Score when it is positive and 0 otherwise.

[d] BH = behavioral health SMI = serious mental illness, SUD = substance use disorder, OUD = opioid use disorder, AUD = alcohol use disorder.

[e] The DxCG score for Models 1 and 2 is the DxCG v4.2 Cotiviti, Inc., Model 312 diagnosis-based concurrent risk score normalized to have mean = 1 in the full population and the DxCG score for Model 3 is the DxCG v5.3 (Cotiviti, Inc., Model 88), normalized to have mean = 1 in the full population.

© 2023 Alcusky MJ et al. *JAMA Network Open.*
