# Supplementary material for: Paying for Medical and Social Complexity in Massachusetts Medicaid
Source: JAMA Netw Open. 2023 Sep 5;6(9):e2332173. doi: 10.1001/jamanetworkopen.2023.32173 (PMC10481227; doi:10.1001/jamanetworkopen.2023.32173)
Supplement: Supplement 2. — Data Sharing Statement [file jamanetwopen-e2332173-s002.pdf]

Data Sharing Statement

Alcusky. Paying for Medical and Social Complexity in Massachusetts Medicaid. *JAMA Netw Open*. Published September 05, 2023. doi:10.1001/jamanetworkopen.2023.32173

Data
Data available: No

Additional Information
Explanation for why data not available: The MassHealth (Massachusetts Medicaid and CHIP) administrative data used for this study included member enrollment, provider characteristics, claims, and encounter files. These data can only be accessed with the permission of MassHealth. Such data may be requested from the Massachusetts Center for Health Information and Analysis by going to https://www.chiamass.gov/chia-data/ or directly at https://www.chiamass.gov/assets/Uploads/data-apps/Non-Government-APCD- Application.pdf.

(See section VIII, Medicaid (MassHealth) Data.)
